# Understanding necrotizing enterocolitis endotypes and acquired intestinal injury phenotypes from a historical and artificial intelligence perspective

**DOI:** 10.3389/fped.2024.1432808

**Published:** 2024-09-27

**Authors:** Alain Cuna, Navin Kumar, Venkatesh Sampath

**Affiliations:** ^1^ Division of Neonatology, Children’s Mercy Kansas City, Kansas City, MO, United States; ^2^ School of Medicine, University of Missouri-Kansas City, Kansas City, MO, United States; ^3^ Division of Neonatology, Hurley Medical Center, Flint, MI, United States

**Keywords:** necrotizing enterocolitis, machine learning, phenotype, endotype, prematurity, neonate

## Abstract

Necrotizing enterocolitis (NEC) remains a devastating disease in preterm and term neonates. Despite significant progress made in understanding NEC pathogenesis over the last 50 years, the inability of current definitions to discriminate the various pathophysiological processes underlying NEC has led to an umbrella term that limits clinical and research progress. In this mini review, we provide a historical perspective on how NEC definitions and pathogenesis have evolved to our current understanding of NEC endotypes. We also discuss how artificial intelligence-based approaches are influencing our knowledge of risk-factors, classification and prognosis of NEC and other neonatal intestinal injury phenotypes.

## Introduction

**Figure 1:**
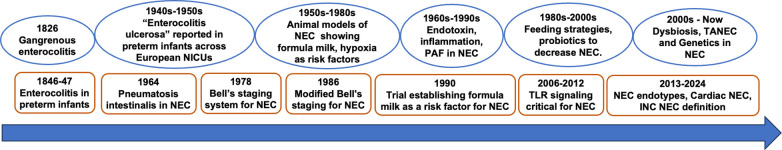
NEC – a brief history in time. INC, International Neonatal Consortium; PAF, platelet activating factor; TANEC, transfusion-associated NEC; TLR, Toll-Like Receptor.

Necrotizing enterocolitis (NEC) is a devastating disease in premature infants with an incidence of 5%–12% in very low birthweight infants (1–4). While less common than in preterm infants, recent studies have also identified risk-factors that predispose full-term neonates to NEC (5–8). Despite significant progress made in understanding NEC pathogenesis over the last 50 years, the inability of current definitions to discriminate the various pathophysiological processes underlying NEC has led to an umbrella term that limits clinical and research progress (9, 10). Further, the lack of precise clinical, biochemical and radiological tools to define NEC has hindered progress in delineating it from different conditions such as septic ileus (9). We propose that identifying endotypes of NEC based on pathophysiology, epidemiology and diagnostic tools will pave the way for precision approaches in preventing and treating NEC (11, 12). In this review, we provide a historical perspective on how NEC definitions and pathogenesis have evolved to our current understanding of NEC endotypes (Figure 1). We also discuss how artificial intelligence (AI)-based approaches are influencing our knowledge of risk-factors, classification and prognosis of NEC endotypes and other neonatal intestinal injury phenotypes.

### NEC: diagnosis and definitions over time

The earliest report of an endotype resembling NEC could be Charles Billard's description 200 years ago from the Hôpital des Enfants Trouvés (13, 14). In his textbook, he describes “gangrenous enterocolitis” in neonates, characterized by abdominal distension, bloody stools, septicemia and death. Autopsy reports showed the ileum was particularly affected with erythema, swelling, ecchymosis, and friability (14). A systematic report of preterm NEC is found in Bednar's description of “entero-colitis” in 25 infants admitted to the Vienna hospital for foundlings between 1846 and 1847, 7 of whom were premature, with majority developing disease between 3 and 30 days of life. In 20 infants who died, autopsy showed evidence of necrosis, gangrene and hemorrhage, very similar to current descriptions of severe advanced NEC (15). In several neonatal intensive care units established across Europe to care for preterm infants between 1910 and 1940s, a NEC-like disease with pathological features including intestinal perforation in lethal cases is poignantly described (16, 17). Between 1948 and 1950, Schmidt and Kaiser et al. described “enterocolitis ulcerosa” in 85 mostly breast-fed preterm infants in Graz, Germany (18, 19). They accurately documented the ileocecal involvement, peritonitis, and perforation and speculated the role of a specific pathogen. Apart from the lack of corroborative radiological, cytological and bacteriology evidence, these physicians established the pathology of NEC and identified the at-risk preterm infant population. These studies did not delve into the pathophysiology of NEC, but hinted at infectious etiology, including viruses.

While pneumatosis intestinalis had been reported in NEC before, it was Berdon in 1964, who described the entire spectrum of what has become the *signe qua non* of NEC diagnosis (20–22). Bell et al. in 1978 proposed the first systematic classification of NEC, grading it from stage I to III based on clinical signs, biochemical markers, radiological signs and disease severity (23). This significant advance enabled consistency among clinicians and researchers to classify NEC more accurately than before, and also provided severity-based treatment guidelines. Around the time Bell et al. classified NEC, several investigators using animal and human studies suggested that hypoxia, formula feeding, speed of feed advancement and infection were risk factors for NEC in the late preterm population (24–26). Walsh and Kliegman in 1986 modified this classification to 6 categories with two subcategories for stage I, II and III (10). In their classical paper, they also summarized the existing thoughts on pathogenesis of NEC indicating the potential roles for direct intestinal infections, bacterial overgrowth, formula feeding, milk intolerance, ischemia, hypertonic enteral supplements in causing mucosal injury and inflammation in an immature gut (10). Interestingly, their summary hinted at multi-factorial causation and a broad spectrum of mechanisms underlying NEC evolution. While several other definitions including the Vermont Oxford Network, Centers of Disease Control and Prevention and the UK Neonatal Collaborative NEC Study group among others have defined NEC, Bell's staging and modified Bell's staging are still the most commonly accepted definitions for NEC (12). From mid-1980s to the current era, several investigators have unraveled the role of dysregulated Toll Like receptor 4 (TLR4) signaling, gut microbiome and dysbiosis, inflammatory mediators and genetic predisposition in NEC (1, 4, 27–35).

Recognizing the limitation of the Bell's criteria in not recognizing spontaneous ileal perforation (SIP), differentiating NEC in term infants vs. preterm infants, and standardizing definitions for research purposes, the International Neonatal Consortium NEC definition groups proposed to classify NEC based on gestational-age, timing of onset of disease, one of the two clinical signs (hematochezia and abdominal distension), and radiological evidence (9). These criteria demarcate NEC that develops in preterm infants from NEC in term infants, and also distinguishes SIP and septic ileus from NEC in preterm infants. While these criteria do address some of the short comings of the previous NEC definitions, it is agnostic with respect to the different pathogenic mechanisms and the resulting endotypes. In the subsequent paragraphs, we will briefly review current understanding of NEC and NEC-like intestinal injury endotypes in preterm and term infants focusing on differences in the pathogenic mechanisms, timing of onset, distinguishing features, and prognosis. A summary of different NEC endotypes is presented in Table 1.

**Table 1:** Summary of different endotypes of necrotizing enterocolitis.

	Clinical presentation	Age/Timing of presentation	Proposed mechanism of injury
Classical NEC	Sudden onset of feeding intolerance, abdominal distention, and bloody stools with systemic signs of illness	Preterm infants, typically occurring between 2 and 6 weeks after birth, around 28–32 weeks postmenstrual age	Deviant host-microbiota interactions in the immature gut triggering excessive intestinal inflammation. Other factors include genetic predisposition and formula-feeding.
Transfusion-associated NEC	Symptoms of classical NEC after packed red blood cell transfusion in otherwise stable preterm infant with established feeding	Preterm infants, within 48–72 h of receiving packed red blood cell transfusion	Ischemia-hypoxemia from chronic anemic state followed by reperfusion injury from transfusion; factors in transfused blood triggering excessive intestinal inflammation
Virus-associated NEC	Gastrointestinal symptoms such as emesis, diarrhea, feeding intolerance ± systemic signs such as fever, apnea, lethargy, or irritability	Preterm infants, occurring in clusters coinciding with peak seasons of viral transmission. Timing variable.	Direct invasion of virus into intestinal epithelial cells leading to intestinal injury and inflammation
Cardiac NEC	Signs of NEC occurring in infant with congenital heart disease, especially lesions with ductal-dependent systemic blood flow	Term infants, typically occurring in the first two weeks of life and/or post-surgical repair, but depends on feeding patterns	Mesenteric ischemia ± reperfusion injury in the setting of cardiac lesion that disrupts systemic perfusion
NEC associated with congenital intestinal anomalies	Signs of NEC occurring in infants with gastrointestinal anomalies such as gastroschisis and Hirschsprung's disease	Term and late-preterm infants, timing is highly variable, but typically in the first weeks of life and/or post-surgical repair	Structural and functional defects in the vasculature or mucosa related to underlying gastrointestinal anomaly that compromise circulation, barrier function and motility
NEC associated with impaired mesenteric blood flow	NEC associated with conditions that could impair mesenteric blood flow such as perinatal asphyxia or polycythemia	Term infants, typically occurring in the first week of life	Combination of mesenteric ischemia ± physiologic demands of feeding
Spontaneous intestinal perforation	Distinct entity from NEC presenting as abdominal distention, associated with exposure to hydrocortisone and indomethacin	Extremely preterm infants, typically occurring in the first 10 days of life with minimal or no feeds initiated	Focal intestinal necrosis of terminal ileum thought to be related to bowel wall ischemia or deficiency of muscularis propria
Cow milk protein allergy	Bloody stools with no systemic signs of illness that resolves with eliminating cow milk protein in diet	Term and preterm infants, occurring much later after several weeks of established feeds	Non-IgE mediated allergic reaction to cow milk protein resulting in intestinal inflammation

### Endotypes of NEC in preterm infants

#### Classical NEC

The most common endotype of NEC – coined “classical NEC” – occurs in preterm infants. The onset of presentation of classical NEC has an inverse relationship with gestational age (36), often occuring in the 28–32 week postmenstrual age with sudden onset of feeding intolerance, abdominal distention, and bloody stools that can rapidly progress towards intestinal perforation, peritonitis, and multi-organ dysfunction (4). The presence of pneumatosis intestinalis and/or portal venous gas on imaging is diagnostic of the disease, while free air heralds intestinal perforation requiring surgery (37). A gasless abdomen, or fixed, dilated loops – defined by persistent location and configuration for more than 24 h – are also a concerning imaging finding for NEC (38, 39).

While the pathogenesis of classical NEC is multifactorial, prematurity remains its single most important risk factor, with NEC incidence rising as gestational age and birth weight decrease. The immature preterm gut is structurally and functionally underdeveloped, with decreased mucosal integrity, reduced motility, and impaired barrier function. Preterm infants also possess an immature immune system that predisposes them to aberrant inflammatory responses. Experimental studies reveal excessive Toll-like receptor (TLR) activation as a key pathway that drives intestinal inflammation in NEC (40). The other major player in classical NEC pathogenesis is the gut microbiota. Dysbiosis – driven by formula-feeding, antibiotic exposure, and perinatal stress – can induce aberrant inflammation in the preterm gut causing mucosal injury, translocation of bacteria into the circulation and subsequent multi-organ dysfunction (1, 29). Conversely, factors that promote a healthy gut microbiome – such as breastmilk, avoidance of prolonged antibiotics, and probiotics – decreases the risk of NEC (41–44).

Despite these advances in our understanding, classical NEC remains a complex disease with significant morbidity and mortality risks. For instance, NEC continues to occur despite avoidance of formula-feeding, exclusive use of human breastmilk, probiotics, and judicious antibiotic stewardship. It remains a leading cause of mortality, especially in infants with extensive intestinal necrosis requiring surgery. Survivors of NEC are also at increased risk for complications including strictures, short gut syndrome, growth failure, and neurodevelopmental impairments.

#### Transfusion associated NEC (TANEC)

Another endotype of NEC in preterm infants is TANEC or transfusion related acute gut injury. TANEC often develops much later than classical NEC – after the 4th or 5th week of life – in otherwise stable preterm infants who have been established on enteral feeds for several weeks (45). Infants who develop TANEC have chronic anemia for several weeks, and symptoms of NEC often are evident within 48 h after packed red blood cell transfusion (45).

The exact pathogenesis of TANEC remains unknown. One proposed mechanism is that chronic anemia could mimic a state of ischemia-hypoxia in the mesenteric bed, and transfusion could trigger a reperfusion injury of previously ischemic intestinal tissue (46, 47). The generation of reactive oxygen species with reperfusion injury, combined with physiological demads of feeding, could be sufficient to cause mucosal damage and compromise the intestinal barrier, leading to TANEC. Based on this pathophysiology, withholding feeds around transfusion has been adopted by some to prevent TANEC (48), although good-quality evidence supporting this practice remains lacking (49). Another proposed mechanism is that intestinal injury arises from hemolytic factors in the transfused blood. In an experimental model of TANEC, free hemoglobin and heme in packed red blood cells were shown to activate monocytes and macrophages in the intestine, triggering excessive TLR inflammation and NEC (50). Interestingly, the Transfusion of Prematurity trial did not show differences in NEC rates among extremely preterm infants randomized to high vs. low transfusion thresholds, and a secondary analyses showed no temporal relationship between red blood cell transfusion and NEC (51, 52).

TANEC poses significant morbidity and mortality risks for preterm infants. In one meta-analysis, TANEC had higher odds of mortality compared to classical NEC (53). In contrast, a study using the Canadian Neonatal Network database found no significant differences in mortality and morbidities between TANEC and classical NEC (54).

#### Viral infections and NEC

Gastroenteritis caused by viral pathogens can mimic NEC (55, 56). Rotavirus (57, 58), cytomegalovirus (59), norovirus (60), astrovirus (61, 62), and enterovirus have been implicated in neonatal gastroenteritis (63). As viral infections are typically not considered in the differential diagnosis of NEC, a high index of suspicion is required. A viral cause is suspected when NEC occurs in clusters, coinciding with peak seasons of viral transmission (55, 64, 65). While clinical presentation can vary, most cases present similarly as classical NEC with gastrointestinal symptoms such as abdominal distention, feeding intolerance, bilious emesis, and bloody stools. Some infants present with systemic signs such as fever, apnea, lethargy, or irritability (e.g., norovirus); while others present with extra-intestinal manifestations such as respiratory symptoms (e.g., enterovirus) or hepatic dysfunction (e.g., cytomegalovirus). Early recognition of viral NEC could limit the use of antibiotics and direct appropriate anti-viral treatment, such as ganciclovir for cytomegalovirus.

The pathogenesis of viral NEC includes direct invasion of virus into intestinal epithelial cells, leading to cellular injury, disruption of tight junctions, and loss of barrier function. Viral infection also triggers an inflammatory response which can mimic classical NEC. Nevertheless, viral NEC tends to have a more insidious onset and a less fulminant clinical course compared to classical NEC, which often presents with rapid progression.

### Endotypes of NEC in term infants

Term infants can also develop NEC, although the incidence is much less than in preterm infants. NEC in term infants typically presents earlier than preterm NEC, with average age of onset in the 1st week of life (5, 66). Term NEC is also typically secondary to other underlying disease processes, most notably congenital heart disease (67).

#### Cardiac NEC

The incidence of NEC in term infants with congenital heart disease is about 2%–6%. The highest risk seems to occurr in conditions with ductal-dependent systemic blood flow such as hypoplastic left heart disease (68–70), although other lesions such as truncus arteriosus and common ventricle have been reported (71). Some studies suggest that the colon is significantly more involved in cardiac NEC (6), but others indicate the small intestine remains the primary location (72).

The pathophysiology of cardiac NEC is thought to be from mesenteric ischemia caused by anatomic lesions that disrupt systemic perfusion during diastole (73). Reperfusion injury following ischemia could also play a major role, particularly in infants who remain at increased risk for NEC even after surgical correction. Other mechanisms include hemodynamic changes while on cardiopulmonary bypass during surgery, or from medications such as vasopressin and opiates post-surgery (74–76). Because feeding can alter gastrointestinal perfusion and hemodynamics, there is often hesitancy to feed infants with cardiac disease, despite the absence of high-quality evidence (77).

Infants with cardiac NEC can have poor outcomes despite its occurrence predominantly in term infants. In one study, mortality rates were higher in infants with cardiac NEC compared to non-cardiac NEC (38% vs. 27%) (78). Prolonged hospital stay, mechanical ventilation, and parenteral nutrition were also noted (70, 71, 74). Although less common, NEC associated with severe congenital heart disease has also been reported in preterm infants, showing higher mortality compared to NEC in preterm infants without congenital heart disease (79).

#### Gastrointestinal anomalies and NEC

Term and late-preterm infants with gastrointestinal anomalies – such as intestinal atresia, malrotation with volvulus, Hirschsprung's disease, gastroschisis, and omphalocele – are also at increased risk of NEC (80–82). In particular, NEC has been reported to occur in up to 20% of infants with gastroschisis (81, 82). The pathogenesis of NEC in these conditions is related to structural and functional anomalies of the intestine including intestinal obstruction, dysmotility, and vascular compromise. The anatomical complexity of gastrointestinal anomalies can increase morbidity and mortality of these infants who also develop NEC, as the presence of gastrointestinal anomalies would typically require surgical repair of the underlying anatomical defect.

#### Other conditions associated with term NEC

Other conditions that could impair mesenteric blood flow or result in mesenteric ischemia – such as perinatal asphyxia, polycythemia, and septic shock – have been reported in term infants with NEC (67). In a review of term NEC cases at Intermountain Health, all cases of term NEC occurred in infants with gavage feeding, overfeeding, and/or feeding with formula (83). Moreover, overfeeding has been shown to be sufficient to elicit NEC injury in a mouse model (84). Thus, a possible unifying hypothesis regarding the pathogenesis of term NEC is the combination of underlying conditions that impair mesenteric blood flow with feeding.

### Acquired intestinal injury phenotypes that mimic NEC

#### Spontaneous intestinal perforation (SIP)

While SIP also develops in extremely preterm infants it is a distinct entity from NEC. SIP tends to occur earlier, even before feeds have been initiated, and is associated with exposure to postnatal indomethacin and hydrocortisone (85, 86). While both can present with abdominal distention, SIP often presents with a bluish discoloration of the abdomen (87). Gross examination of the intestine in SIP reveals the perforation localized to the antimesenteric border of the small intestine with healthy tissue surrounding it. In NEC with perforation, the surrounding bowel is not healthy (88). The absence of bowel injury allows for SIP to have simpler surgical treatment with either peritoneal drainage, direct repair, or resection and primary anastomosis (87). In contrast, surgical NEC often requires resection of injured tissue with creation of stomas. Mortality from SIP is also lower compared to surgical NEC (89), but surprisingly morbidity and neurodevelopmental outcomes were not better (90, 91).

#### Cow milk protein allergy (CMPA)

CMPA, also known as Food Protein-Induced Enterocolitis or FPIES, can present in both term and preterm infants with bloody stools and is thus an important differential for NEC (92). CMPA tends to occur much later, after several weeks of established feeds with formula or breastmilk that contains cow's milk protein (8). Severe cases can present with *pneumatosis intestinalis*, but otherwise do not progress towards systemic and multi-organ dysfunction (93). It can be difficult to distinguish CMPA from NEC. Typically, CMPA is suspected when reintroduction of feeds that contain cow-milk protein leads to “reoccurrence” of NEC-like symptoms (94). The pathogenesis of CMPA is non-IgE mediated allergic reaction to cow milk protein resulting in infiltration of intestinal mucosa with eosinophils, lymphocytes, and mast cells (95). In contrast to NEC, CMPA is a benign condition with low morbidity and typically improves with elimination of cow's milk protein from the infant's or mothers diet.

### Machine learning in NEC risk identification, diagnosis, and endotype classification

While clinicians have strived to define NEC more precisely and differentiate it from conditions that mimic NEC, these classifications still rely on prior conditioned learning (11). The potential for artificial intelligence (AI)-based approaches to classify in an unbiased fashion disease endotypes has resulted in several studies applying machine learning (ML) to classify NEC. AI is particularly useful in analyzing large datasets to detect intricate patterns not discoverable to human intelligence, and holds promise to improve diagnosis, classification, and management of acquired intestinal injury (96). When AI is utilized to analyze data within frameworks such as specific diagnosis/labels, known risk factors, or pre-determined outcomes it is commonly known as *supervised* ML. When AI is used to analyze data without labels or pre-determined framework/outcome, it is considered *unsupervised* analysis. In Table 2, a collection of ML models is depicted, along with a concise explanation and their contextual applications in NEC research.Through these models, patterns of disease in NEC that were not readily appreciated can be discovered (97).

**Table 2:** Summary of different machine learning models used in NEC research.

ML model	Category	Contextual applications in NEC research	Description
Artificial Neural Network (ANN)	Supervised/Unsupervised	Used to predict NEC risk based on clinical features and patient data.	Comprises interconnected nodes that simulate neural processes.
Deep Learning (DL)	Supervised/Unsupervised	Used for examining imaging data related to NEC with pixel-level analysis.	An effective multi-layer variant of ANN designed for handling large datasets.
Logistic regression	Supervised	Used to classify the presence or absence of NEC through binary classification.	A probability-based statistical approach for binary outcomes.
Decision tree	Supervised	Used for risk stratification and outcome prediction in neonatal patients.	A flow-chart-like structure that facilitates decision-making processes.
Random forest	Supervised	Utilized for the identification of significant risk factors associated with NEC.	Using a combination of multiple decision trees to improve accuracy.
Naïve bayes	Supervised	Employed for the categorization of neonatal patient data according to predetermined characteristics.	A probabilistic model that relies on Bayes’ theorem and assumes independence.
Support Vector Machine (SVM)	Supervised	Used to distinguish between neonates at risk of NEC and those who are not.	A classification method that identifies the optimal hyperplane in high-dimensional space.
Back-propagation neural network	Supervised	Utilized to train ANN models specifically for NEC outcomes.	An algorithm that updates neural network weights through error feedback.

Apart from prematurity, formula milk feeding, African American race (either as a social determinant of health or genetic risk), and potentially genetic factors, risk factors that are consistently associated with NEC remain unclear (1, 98, 99). Recently, Mueller et al. (100) used artificial neural networks to identify small for gestational age and use of artificial ventilation as additional risk factors for NEC. The utilization of continuous vital signs data has proven to be highly beneficial in diagnosing life-threatening diseases and improving outcomes for sepsis and other conditions in preterm infants (101). Vital signs data can also be leveraged using ML techniques to accurately predict NEC. Doheny et al. (102) analyzed the high frequency component of heart rate variability, an indicator of baseline vagal tone, in 70 preterm infants. The authors found that decreased vagal tone was a highly accurate predictor of NEC. ML can be used to identify white blood cell patterns to diagnose and prognosticate NEC. In a retrospective cohort of 246 infants, Pantalone et al. (103) reported that the onset of NEC in more mature infants (born after 33 weeks) was associated with lower neutrophil counts at diagnosis compared to controls. In less mature infants, a sharp decrease in monocytes and lymphocytes, as well as an elevation in bands at the time of diagnosis, predicted surgical intervention. The type of ML algorithm employed can also affect the findings as shown by Cho et al. (104) who compared the ability of several ML models to predict NEC. The study dataset consisted of over 10,000 very low birthweight infants and 74 variables, including environmental factors. Logistic regression and random forest (RF) exhibited superior performance achieving accuracy rates of 0.93, compared to artificial neural network, decision tree, naïve Bayes, and support vector machine methods. Birth weight, maternal age, gestational age, sepsis, male sex, and environmental factors such as ambient temperature were highlighted as key predictors, among others.

ML models have been used to predict intestinal perforations in NEC patients (NEC-IP). Using a Back-propagation neural network model, Irles et al. (105) identified that platelet counts, neutrophil counts, intubation, birth weight and arterial blood gas parameters can accurately predict NEC-IP. Recent ML studies have also demonstrated its utility to differentiate SIP from NEC-IP. Models such as random forest, ridge logistic regression model (106), and artificial neural network (107) have shown accuracy rates higher than 90% in differentiating these two conditions even before surgery, which can help guide optimal management strategies. Recent studies have also used ML models to predict need for surgical intervention in NEC (108, 109).

Researchers have also examined stool microbiome and metabolomic data using unsupervised ML algorithms to predict NEC (97). Notably, Lin et al. (110) used a multiple instance learning (MIL) architecture for predicting NEC based on stool microbiota data. Through incorporating past data with analysis of crucial bacterial taxa, this approach achieved timely and precise prediction of NEC risk, with an average lead time of 8.3 days. Their RF model emphasized the importance of Firmicutes, Proteobacteria and Enterobacteriaceae in NEC prediction with high level of sensitivity and specificity, thus emphasizing its potential in enabling personalized risk assessment and disease prevention (110). Other studies examined stool metabolomic (111) and urine peptides data (112) to find specific patterns to predict NEC. Recent endeavors have undertaken an unbiased assessment of distinct patterns of acquired intestinal injuries in preterm infants. By utilizing an unsupervised hierarchical clustering algorithm, Gipson et al. (113) successfully identified five distinct clusters of acquired neonatal intestinal injuries from a sample of 183 infants who experienced 210 episodes of such injuries. These clusters were classified as (1) low mortality, (2) immature with high mortality, (3) mature with inflammation, (4) late injury at full feeds, and (5) late injury with intestinal necrosis. These studies provide encouraging data for improving the prediction, accurate diagnosis and prognosis of NEC and other intestinal phenotypes that mimic NEC. One limitation of current AI studies includes limited data from single center design, smaller cohorts, and non-uniformity of variables used for analysis. As the accuracy and reliability of AI models rely solely on the quality of the input provided, reliability and generalizability of these models can be enhanced by using standardized datasets across multiple centers. Another important limitation is the complex and multi-factorial nature of NEC pathogenesis, which makes capturing all relevant variables and their interactions in an AI model challenging. AI models would also need to be continuously updated to incorporate new insights and research findings as our understanding of NEC evolves.

## Conclusion

While our understanding of NEC has evolved over time from a clinical/pathological description to a better understanding of pathophysiology and NEC endotypes, only limited progress has been made in in differentiating classical NEC from endotypes that mimic it but have different etiologies and prognosis. We speculate that characterizing endotypes of NEC based on pathophysiology, clinical variables and radiological/biochemical tests using traditional clustering methods augmented by machine learning (ML) is important for precision approaches directed at disease prevention and management of NEC and acquired intestinal injury phenotypes in neonates.
